# The neighborhood social environment and body mass index among youth: a mediation analysis

**DOI:** 10.1186/1479-5868-9-31

**Published:** 2012-03-20

**Authors:** Jenny Veitch, Maartje M van Stralen, Mai JM Chinapaw, Saskia J te Velde, David Crawford, Jo Salmon, Anna Timperio

**Affiliations:** 1 Centre for Physical Activity and Nutrition Research, Deakin University, 221 Burwood Highway, Burwood, 3125, Australia; 2 EMGO Institute for Health and Care Research and Department of Public and Occupational Health, VU University Medical Center, Van der Boechorstraat 7, 1081BT, Amsterdam, the Netherlands; 3 EMGO Institute for Health and Care Research and Department of Epidemiology and Biostatistics, VU University Medical Center, Van der Boechorstraat 7, 1081BT, Amsterdam, the Netherlands

**Keywords:** Neighborhood, Social networks, Social cohesion, Youth, Physical activity, Sedentary behavior, Overweight, Mediation

## Abstract

**Background:**

This study aimed to examine associations between aspects of the neighborhood social environment and body mass index (BMI) in youth both cross-sectionally and prospectively; and whether this association was mediated by physical activity, screen-time and sedentary time.

**Methods:**

Data were collected in 2004 and 2006 in high and low socio-economic areas of Melbourne, Australia. In 2004, 185 children aged 8-9 years (47% boys) and 359 children aged 13-15 years (45% boys) participated. Parents reported their perceptions of aspects of the social environment (i.e. social networks and social trust/cohesion), and physical activity (i.e. time spent outdoors by their children; and their younger children's walking and cycling trips) and screen-time (i.e. TV viewing, computer use). The older children self-reported their walking and cycling trips and their screen-time. All children wore an accelerometer to objectively assess outside-school hours moderate- to-vigorous physical activity and sedentary time. BMI was calculated from height and weight measured in 2004 and 2006. Multilevel linear regression analyses were conducted to examine associations between the social environment and BMI. Mediation analyses using the products of coefficient method were conducted to determine whether associations between the social environment and BMI were mediated by the time spent in a range of physical activity and sedentary behaviors.

**Results:**

Cross-sectional and prospective regression analyses showed that a more positive social network and higher social trust/cohesion was related to lower BMI among children. There was no evidence that time spent in physical activity or sedentary behaviors mediated this relation, despite significant associations between social networks and screen-time and between screen-time and BMI.

**Conclusions:**

The findings suggest that the neighborhood social environment may be important for preventing overweight and obesity in children. Further research investigating the mechanisms through which the neighborhood social environment exerts its effect on BMI is needed.

## Background

Child and adolescent overweight and obesity represents a significant global public health burden, with a high and increasing prevalence in many developed countries, serious health consequences both during childhood and adolescence and in adulthood, and increased likelihood of remaining overweight and obese as adults [1,2]. Prevention of overweight in childhood and adolescence is therefore critical and a thorough understanding of influences on the development of overweight is needed. Ecological models suggest that childhood overweight is the result of a complex array of factors operating at multiple levels, from individual to family to school, neighborhood, community and policy influences [3]. Recently, the importance of neighborhood environments for promoting or hindering youth physical activity and active transport has received considerable attention [4] and many studies have also examined the influence of neighborhood environments on youth overweight [5-7]. However, most of those studies focus on the built environment. Studies of social factors within neighborhoods and youth overweight are less common and have generally been limited to an examination of perceived safety and crime [7]. Constructs such as neighborhood social cohesion and social networks are rarely studied. However, it has been suggested that socially cohesive neighborhoods may influence youth physical activity by facilitative enforcement of healthy norms, community awareness of programs and facilities, collective action to improve the local area and limiting crime and disorder [8].

Associations between social capital, trust or cohesion and physical activity have been observed cross-sectionally [9-11] and longitudinally [8]. Positive associations between social ties or networks (i.e. area has lots of children, friends live nearby, know neighbors) have also been reported for walking and cycling cross-sectionally [12,13] and longitudinally [14]. However, few studies have examined such social constructs in relation to overweight and all are cross-sectional [10,15-17]. Those studies found inverse associations between the social environment and overweight, but each examined different aspects of the social environment, including social trust [10], informal social control [16] and collective efficacy [17].

Although there is some evidence that neighborhood social environments are associated with overweight in children, this evidence is based mainly on cross-sectional samples, with little consistency in measures of the social environment. In addition, no studies have considered pathways through which the social environment may operate to influence childhood obesity. Physical activity, time outdoors, sedentary time (objectively assessed time spent sedentary), and screen-time (watching TV, using the computer and playing electronic games) are likely to be on the causal pathway between the social environment and overweight as these behaviors have been shown to be associated with body mass index (BMI) in youth [18,19] and are also likely to be influenced by the social environment [20]. Two key aspects within the social environment are social networks (i.e. how well neighbours know each other and whether there are other children around to play with) and social trust and cohesion between neighbours. Therefore, this study aimed to examine: 1) cross-sectional and prospective associations between neighborhood social networks and social trust/cohesion and BMI among youth; and 2) to determine whether these associations were mediated by the time spent in a range of physical activity behaviors, screen-time and total sedentary time. It was hypothesized that children who lived in neighborhoods with strong social networks and cohesion would spend less time sedentary and in front of screens and more time in moderate- to-vigorous physical activity (MVPA), more time outdoors, and would take more walking and cycling trips in their neighborhood, which would reduce the likelihood of overweight and obesity.

## Methods

The data in this study were drawn from the first and second follow-up data collection waves of the 'Children Living in Active Neighborhoods (CLAN)' study [21,22]. At baseline (2001), 1210 families of 5-6 year old and 10-12 year old children were recruited from 19 state schools in high (n = 10) and low (n = 9) socio-economic areas of metropolitan Melbourne (44% response rate from those individuals invited). Schools were selected using stratified random sampling proportionate to school size, based on the Australian Bureau of Statistics' Socioeconomic Indices for Areas [23]. Recruitment of the baseline sample has been reported previously [21,22]. Families who indicated that they were interested in participating in further research were re-contacted in 2004 (n = 587). The sample comprised 189 children aged 8-9 years and 398 children aged 13-15 years. This process was repeated in 2006 (n = 487). The present study includes both the younger and older cohorts of children and examines data from 2004 and 2006. Only participants with complete outcome variable (BMI) data in 2004 (n = 544) and 2006 (n = 430) respectively were included in the cross-sectional and prospective analyses. Participants included in the present study did not differ significantly from those who did not continue past baseline or who had missing BMI data with regards to the sex of the child or the age of the parent completing the survey. There was, however, a significant difference with regard to BMI. Those included in the analyses had a lower BMI than those who did not continue past baseline or with missing BMI data (18.3 vs 19.4 kg/m^2^, *p *< 0.05).

More female carers of children with complete BMI data also reported to have a high educational level at baseline compared with females who did not participate in follow-up data collections (41.2% vs 30.5% had high level of education, *p *< 0.05). Written consent was required from parents as well as from the older cohort of children. The Deakin University Human Research Ethics Committee, the Victorian Department of Education and the Catholic Education Office provided ethical approval.

### Measures

In 2004, questionnaires were completed at home by parents (of both age groups of children) and also by the older cohort of children. Parents self-reported their perceptions of aspects of the neighborhood social environment (i.e. social network and social trust/cohesion), and proxy-reported the time spent outdoors by their children. Parents also proxy-reported the number of walking and cycling trips in their neighborhood and screen-time for the younger cohort of children. The older children self-reported their walking and cycling trips and their screen-time. In addition, in order to objectively measure MVPA and sedentary time all children wore an accelerometer for eight days and BMI was calculated from height and weight measured in 2004 and 2006.

#### Socio-demographics

The parent questionnaire assessed age, sex, and highest level of maternal education; collapsed into low (did not complete high school), medium (high school or technical or trade certificate) or high (University or tertiary qualification). Maternal education is presented as a proxy for socioeconomic position [24].

#### Body mass index

In 2004 and 2006, children's height and weight were measured at school by trained research assistants using digital scales and a portable stadiometer. For a small number of children (6.8% in 2004, 5.6% in 2006) parents recorded these measures at home. BMI (kg/m^2^) was calculated. For descriptive purposes only (see Table 1), international age- and gender-specific cut-points [25] were applied to BMI to define three weight status categories; healthy weight, overweight and obese.

**Table 1:** Descriptive statistics of the study sample (2004)

Demographics	Mean (SD),%
N	544
Age (years), mean (SD)	12.6 (2.6)
Sex (boys)%	47.2
Maternal education	
Low (%)	22.6
Medium (%)	33.8
High (%)	43.6
BMI 2004, mean (SD)	20.5 (3.99)
Weight status 2004	
Healthy weight (%)	72.4
Overweight (%)	20.8
Obese (%)	6.8
**Sedentary behaviors**	
Screen-time (mins/day), mean (SD)	179.9 (98.1)
Percentage of outside-school hours sedentary time, mean (SD)	21.8 (5.2)
**Physically active behaviors**	
Time spent outdoors (mins/day), mean (SD)	99.9 (61.4)
Percentage of outside-school hours MVPA, mean (SD)	5.5 (3.5)
Number of walking/cycling trips/week, mean (SD)	16.0 (18.0)
Neighborhood social environment variables*	
Social network score (range 1-5), mean (SD)	3.5 (0.9)
Social trust/cohesion score (range 1-5), mean (SD)	3.6 (0.7)

* Higher score indicates better social networks or social cohesion, respectively

### Neighborhood social environment variables

#### Social network

Parents reported agreement with the statements: 'I know many people in this neighborhood'; 'My child has many friends in this neighborhood'; and 'There are not many other children around for my child to play with (reverse scored)' on a scale of 1-5, with one representing strongly disagree and five representing strongly agree. Scores for these three items were combined and averaged to form a 'social network' score, with one representing weaker and five representing stronger social networks. Internal reliability was assessed using Cronbach's alpha (0.67) and test-retest reliability was assessed in 2004 using Intra-class correlation coefficients (ICC = 0.83) based on a separate sample of 97 parents.

#### Social trust/cohesion

Parents also reported agreement with five statements related to neighborhood trust/cohesion [26]: 'People around my neighborhood are willing to help their neighbors'; 'This is a close-knit neighborhood'; 'People in this neighborhood can be trusted'; 'People in this neighborhood generally don't get along (reverse scored)'; and 'People in this neighborhood do not share the same values (reverse scored)'. Responses were provided on the same scale as the social network items and averaged to compute a 'social trust/cohesion' score (Cronbach's alpha = 0.83; ICC = 0.76).

### Potential mediating variables

#### Screen-time

Parents of the younger children proxy-reported and the older children self-reported the total amount of time in hours/minutes usually spent; 1) watching TV/Videos/DVD's, 2) playing Playstation/Nintendo/computer games, and 3) using the computer/internet Monday to Friday and on the weekend [27]. Responses were summed and divided by seven to create average duration (mins/day) of 'screen-time' (ICC = 0.61).

#### Time spent outdoors

Parents of both the older and younger children were asked to report how many hours/minutes their child usually spends outside during a typical week after school separately for warmer (school terms 1 and 4) and cooler (school terms 2 and 3) months. These two questions were repeated for 'a typical weekend'. The total minutes per day spent outside on weekdays and weekend days was calculated and divided by seven to calculate the average minutes per day spent outside in warmer and cooler months. Responses for the two seasonal periods were summed and divided by two to calculate total 'time spent outdoors' in minutes/day. Test re-test reliability previously reported as (ICC = 0.54) [28].

#### Number of walking/cycling trips in the neighborhood

Parents of the younger children proxy-reported and the older children self-reported how often their child/they usually walk or rides a bike to bike/walking tracks, friends' house, parks/ovals/playgrounds, the post box, public transport, school, shops, or sport venues. Seven response categories were provided (scores assigned are presented in parentheses): it's not within walking/riding distance (0); never/rarely (0); less than once per week (0.5); 1-2 times per week (1.5); 3-4 times per week (3.5); 5-6 times per week (5.5); and daily (7). The scores were summed to compute total number of weekly walking or cycling trips to all destinations (possible range of 0-56 trips). Test re-test reliability previously reported as (ICC = 0.50) [29].

#### Sedentary and physical activity time outside-school hours

Children were asked to wear an accelerometer (Manufacturing Technologies, Inc [MTI] Model 7164; Actigraph, Inc, Florida, USA) attached to an elasticized belt at hip-level for eight consecutive days, removing it only for sleeping, showering or swimming [21]. Movement counts were recorded in 1-minute epochs. Accelerometer data files were downloaded and entered into a data-reduction program. A universal threshold of < 100 counts/min was applied to define sedentary time [30] and the equivalent of 4.0 METS [31] using Freedson age-adjusted cut-points was applied to define MVPA [32]. The data reduction program calculated the total time the accelerometer was worn each day from first and last counts within a 24-hour waking period, and non-wearing periods or 10-minute bouts or longer of zero movement counts were subtracted [33]. On weekdays, sedentary and MVPA time during the before-school (6 am until first school bell), after-school (last school bell until 6 pm) and evening (6-9 pm) periods were summed and expressed as a proportion of total wear time for that day. Proportion of time sedentary and in MVPA was averaged across weekdays for valid days (defined as > 50% wear-time during the after-school period). Time spent at school was not included in the objective measure as this study focuses on the neighborhood environment and leisure-time physical activity and sedentary behaviors. Proportion of time spent sedentary and in MVPA on weekends was computed based on total wear time and averaged across valid weekend days (defined as at least eight hours of wear time) [34]. Only children who recorded at least eight hours of wear-time daily and 50% of wear-time in the after-school period on at least three weekdays and recorded at least eight hours of wear-time on at least one weekend day were included. The average proportions of the day spent sedentary and in MVPA outside-school hours on weekdays were multiplied by five, and the average proportions on weekends were multiplied by two. These values were summed and divided by seven to compute the average proportion of time spent sedentary and in MVPA outside-school hours.

### Statistical analyses

To examine the cross-sectional (2004 only) and prospective associations between the neighborhood social environment and BMI, and to examine the mediating effects of sedentary and physical activity behaviors on this association, a series of multi-level linear regression analyses (using MLwin version 2.02) were conducted. In the prospective analyses, BMI values measured in 2006 were used as the outcome variable, while the values measured in 2004 were used for the predictor and mediator variables. Two levels were defined in the multi-level regression: 1) student, and 2) school. We checked for possible confounding by sex, socioeconomic status (SES) and age. To test whether SES was a confounder, two indicators of SES were included separately in the main regression analyses: 1) maternal education; and 2) area level SES (based on the score for the Socio-Economic Index for Areas (SEIFA) Index of Relative Socio-Economic Advantage/Disadvantage score for each child's residential postal code). As there was no evidence of confounding, neither of the SES indicators were included in the final models.

**Figure 1:**
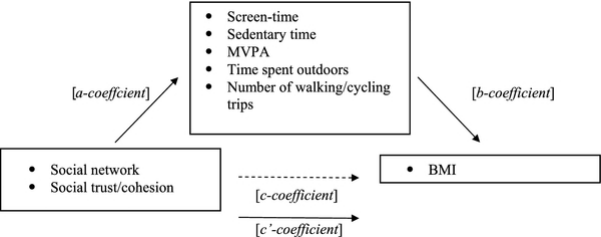
**Conceptual model: Screen-time, sedentary time, MVPA, time spent outdoors and number of walking/cycling trips as mediators of the association between the neighborhood social environment and BMI**.

The cross-sectional and prospective associations between the neighborhood social environment and BMI was calculated (*c-coefficient*/total effect), by regressing the social environmental variable (i.e. social network or social trust/cohesion) and confounders on the outcome variable (i.e. BMI 2004, or BMI 2006) (see Figure 1). The mediated effect of screen-time, sedentary time, MVPA time, time spent outdoors, and the number of walking/cycling trips on the association between neighborhood social environment and BMI, was then calculated using the products of coefficient method [35]. First, the associations between the neighborhood social environment and the potential mediators were calculated using separate regression models (*a-coefficient*). Second, the associations between the potential mediators and BMI, controlling for the neighborhood social environment variables were calculated using separate regression models (*b-coefficient*). This model also provided the direct effect of the neighborhood social environment on BMI, adjusting for potential mediators (*c'-coefficient*). The mediated effect is the product of the a and b coefficient (a*b) and provides an estimate of the relative strength of the mediation effect. The Sobel test was used to assess the statistical significance and 95% confidence intervals of a mediating effect by dividing the products-of coefficients (a*b) by its standard error SE_ab _= √((a^2^*SE_b_^2^) + (b^2^*SE_a_^2^)). In this equation, 'a' stands for the *a-coefficient *and 'b' stands for the *b-coefficient*.

## Results

Table 1 shows the characteristics of the sample and the distribution of all variables. The average age of children in 2004 was 12.6 years (47% boys), and the average age of the parent completing the survey in 2004 was 42.8 years (88% female). Average BMI of children was 20.5 in 2004 and 21.4 in 2006.

### Associations between the social environment and BMI (c-coefficient)

Significant inverse cross-sectional associations were found between social network and BMI and between social trust/cohesion and BMI. Significant inverse associations remained between social network and social trust/cohesion reported in 2004 and BMI in 2006 (Table 2).

**Table 2:** Cross-sectional and prospective associations between the neighborhood social environment and BMI (*c-coefficient*)

Predictor variables	Cross-sectional association between social environment and BMI_2004 _*(c) *(95%CI)	Prospective association between social environment in 2004 and BMI_2006 _*(c) *(95%CI)
Social network score	-0.70 (-1.05, -0.35)	-0.52 (-0.92; -0.13)
Social trust/cohesion score	-0.58 (-1.04, -0.13)	-0.52 (-1.03; -0.003)

95%CI = 95% confidence intervals; all coefficients significant (*p *< 0.05)

Models were adjusted for age and gender

### Associations between the social environment and potential mediators (a-coefficient)

Associations between the neighborhood social environment and the potential mediators for the cross-sectional and prospective samples are shown in Tables 3 and 4.

**Table 3:** Cross-sectional mediation analyses for the association between social environment variables (2004) and BMI (2004) using multi-level linear regression analysis

		BMI in 2004		
	**Association between social environment variables and potential mediators** ***(a) *(95% CI)**	**Association between potential mediators and BMI** ***(b) *(95%CI)**	**Direct effect on BMI adjusting for mediators** ***(c')*** **(95%CI)**	**Mediated effect** (a*b) **(95%CI)**

***Social network***				
Screen-time (mins/day)	**-13.65 (-23.61, -3.68)**	**0.005 (0.001, 0.009)**	**-0.70 (-1.06, -0.34)**	-0.07 (-0.14, 0.005)
% sedentary time	-0.098 (-0.59; 0.40)	0.001 (-0.07; 0.07)	**-0.59 (-0.97; -0.20)**	0.00 (-0.007; 0.007)
% MVPA time	**0.38 (0.10; 0.66)**	-0.074 (-0.21; 0.06)	**-0.56 (-0.95; -0.17)**	-0.03 (-0.08; 0.03)
Time spent outdoors (mins/day)	**13.88 (7.89, 19.87)**	0.002 (-0.004, 0.008)	**-0.73 (-1.08, -0.37)**	0.03 (-0.055, 0.11)
Number of walking/cycling trips (per week)	1.04 (-0.69, 2.76)	0.007 (-0.01, 0.03)	**-0.71 (-1.06, -0.36)**	0.007 (-0.015, 0.029)
***Social trust & cohesion***				
Screen-time (mins/day)	-10.76 (-23.60, 2.09)	**0.005 (0.001, 0.009)**	**-0.58 (-1.04, -0.11)**	-0.054 (-0.13, 0.02)
% sedentary time	0.30 (-0.35; 0.95)	0.003 (-0.07; 0.08)	-0.45 (-0.96; 0.06)	0.001 (-0.02; 0.02)
% MVPA time	-0.055 (-0.42; 0.31)	-0.098 (-0.23; 0.04)	-0.44 (-0.97; 0.06)	0.005 (-0.03; 0.04)
Time spent outdoors (mins/day)	-1.66 (-9.68, 6.36)	-0.000 (-0.006, 0.006)	**-0.48 (-0.95, -0.02)**	0.000 (-0.01, 0.01)
Number of walking/cycling trips (per week)	0.27 (-1.83, 2.37)	0.007 (-0.01, 0.03)	**-0.59 (-1.04, -0.13)**	0002 (-0.01, 0.02)

95%CI = 95% confidence intervals

**BOLD **= significant associations

Models were adjusted for age and gender

**Table 4:** Prospective mediation analyses for the association between social environment variables (2004) and BMI (2006) using multi-level linear regression analysis

		BMI in 2006		
	**Association between social environment variables and potential mediators** ***(a) *(95% CI)**	**Association between potential mediators and BMI*(b) *(95%CI)**	**Direct effect on BMI adjusting for mediators** ***(c')*** **(95%CI)**	**Mediated effect** (a*b) **(95%CI)**
***Social network***				

Screen-time (mins/day)	-9.88 (-20.57, 0.81**)**	**0.004 (0.000; 0.008)**	**-0.52 (-0.92; -0.11)**	-0.040 (-0.097; 0.02)
% sedentary time	0.05 (-0.49; 0.59)	-0.027 (-0.11; 0.06)	-0.41 (-0.84; 0.02)	-0.001 (-0.02; 0.01)
% MVPA time	**0.39 (0.09; 0.69)**	-0.05 (-0.19; 0.10)	-0.39 (-0.82; 0.04)	-0.018 (-0.08; 0.04)
Time spent outdoors (mins/day)	**13.54 (7.10, 19.99)**	0.003 (-0.003; 0.009)	**-0.58 (-0.98; -0.18)**	0.041 (-0.04; 0.12)
Number of walking/cycling trips (per week)	**1.23 (0.03,2.57)**	0.002 (-0.027, 0.031)	**-0.52 (-0.92, -0.13)**	0.003 (-0.036, 0.041)
***Social trust & cohesion***				
Screen-time (mins/day)	-4.75 (-18.66, 9.17)	**0.004 (0.000; 0.008)**	-0.49 (-1.02; 0.04)	-0.019 (-0.08; 0.04)
% sedentary time	0.097 (-0.62; 0.82)	-0.03 (-0.11; 0.06)	-0.37 (-0.95; 0.22)	-0.003 (-0.03; 0.02)
% MVPA time	0.09 (-0.32; 0.49)	-0.07 (-0.22; 0.08)	-0.36 (-0.94; 0.22)	-0.006 (-0.04; 0.02)
Time spent outdoors (mins/day)	1.59 (-7.04, 10.22)	0.001 (-0.005; 0.007)	-0.49 (-1.01; 0.03)	0.002 (-0.01; 0.014)
Number of walking/cycling trips (per week)	0.27 (-1.38, 1.92)	0.0 (-0.03, 0.03)	**-0.52 (-1.03, -0.003)**	0.0 (-0.008, 0.008)

95%CI = 95% confidence intervals

**BOLD **= significant associations

Models were adjusted for age and gender

### Associations between potential mediators and BMI (b-coefficient)

Tables 3 and 4 show associations between the potential mediators and BMI adjusted for the neighborhood social environment factors. Significant positive cross-sectional and prospective associations between self- and proxy-reported screen-time and BMI were found when adjusted for social networks and social trust/cohesion. No other significant associations between any of the potential mediators and BMI were found.

### Effect of potential mediators on association between social environment and BMI (ab-coefficient)

No statistically significant mediated effect of any of the potential mediators was found on the cross-sectional or prospective associations between the social environment variables and BMI (Tables 3 and 4).

## Discussion

This study examined cross-sectional and prospective associations between aspects of the neighborhood social environment and BMI, and examined whether this association was mediated by a range of physical activity and sedentary behaviors. Consistent with previous cross-sectional studies [10,16,17], we found negative associations between neighborhood social networks and social trust/cohesion and BMI, with stronger associations cross-sectionally between social networks and BMI. Interestingly, inverse associations were also found prospectively two years later. This indicates that the more positive the neighborhood social networks and social trust/cohesion in 2004, the lower the children's BMI at that time point and two years later. These findings suggest that these aspects of the neighborhood social environment may be important determinants of overweight and obesity in youth. Maternal and area-level SES did not confound these associations.

In order to explain associations between neighborhood social networks and social trust/cohesion and overweight it is important to examine the associations between social networks and social trust/cohesion and the potential mediators *(a-coefficient) *and the associations between the mediators and BMI *(b-coefficient)*. Our 2004 cross-sectional findings suggest that children who live in a neighborhood with strong perceived social networks tend to spend less time in screen-time, more time in MVPA outside-school hours, and more time outdoors. These results confirm other studies [9,12,13,15] including a study by Franzini et al. that found that the neighborhood social environment was positively associated with self-reported physical activity, which was negatively associated with child obesity [15]. In the current study, no associations were found between social networks and the number of walking/cycling trips or percentage of sedentary time outside-school hours. Social trust/cohesion was not associated with physical activity, screen-time or sedentary time. This indicates that how well people get along with and trust their neighbors may be of less importance to these behaviors than factors relating to social networks, such as children having other children nearby to play with. This is consistent with our previous cross-sectional research that highlighted the importance of social networks to children's outdoor play in the neighborhood [36,37]. Given the inconsistent findings across the measures of the social environment, further research using different measures of aspects of the neighborhood social environment may be warranted.

Through our examination of associations between the potential mediators and BMI *(b-coefficient) *we also identified that children who spent more time in front of screens had a higher BMI both cross-sectionally and two years later. Surprisingly, neither total sedentary time outside-school hours, as assessed by accelerometry, or any of the physical activity behaviors were associated with BMI in any analyses. Previous cross-sectional studies have shown inverse associations between children's objectively measured physical activity and BMI [38], and positive associations between children's screen-time and overweight and obesity [19]. Epstein et al. (2008) demonstrated that changes in screen-time caused changes in adiposity in young children, and this was the result of changes in energy intake rather than physical activity [39]. In addition, some studies suggest displacement of physical activity with screen-time, although these data are equivocal [19]. A small number of studies have examined the relation between objectively-assessed sedentary time and children's overweight and obesity. A cross-sectional study involving over 5,000 12 year old child in the UK found for every hour per day spent sedentary (operationalized as < 200 accelerometer cpm) after adjusting for sex, social factors, sleep, TV time and pubertal status, children were 32% more likely to be obese (body fat was measured by DEXA) [40]. Interestingly, the relationship between sedentary time and obesity was no longer significant once analyses adjusted for MVPA. Consistent with findings from the current study, few other cross-sectional or prospective studies have shown that objectively measured sedentary time is associated with obesity in children and adolescents [41-43].

While it is intuitive that positive social environments are likely to influence weight by providing environments in which youth feel safe enough to walk, cycle, or be active, the findings from this study showed no evidence that the physical activity or sedentary behaviors included in our model mediated the association between neighborhood social networks and social trust/cohesion and BMI. The lack of mediated effect indicates that the social environmental determinants did not exert their effects on BMI via the sedentary and physical activity behaviors assessed. The working mechanisms through which neighborhood social networks and social trust/cohesion exert their effect on BMI are therefore still unclear. Although, the measures of SES available in this study did not confound the associations between the social neighborhood variables and BMI, it is possible that neighborhoods that score high on social environment are neighborhoods with greater levels of socio-economic advantage. It may also be possible that children living in areas with high perceived social environment may have healthier eating patterns, which may explain mediating pathways with BMI. Future research could incorporate children's eating behaviors in addition to physical activity and sedentary behaviors.

The study had some limitations. The neighborhood social environment was limited to the examination of social networks and social trust/cohesion as reported by parents and children may have perceived their social environment differently to their parents. The inclusion of other aspects of the social environment and different ways of measuring social networks and social trust may have also resulted in alternative findings. In addition, parents reported screen-time and walking and cycling trips in the neighborhood for the children in the younger cohort, whereas these behaviors were self-reported by the older cohort. There were also significant differences in BMI and maternal education between those included in the present study and those who did not continue past baseline or who had missing BMI data. Further, due to the sample size it was not possible to analyze data separately by age and sex. It may be that social networks and social cohesion/trust exert a greater influence on physical activity levels of older children who are more likely to be able to walk and cycle in their neighborhood without adult supervision [22]. In addition, other factors known to influence obesity and that might confound the relationship between the neighborhood social environment and obesity (e.g. parental obesity, cultural background/ethnicity, diet and sleep) were not assessed. Future studies may benefit from including dietary intake data as a potential mediating variable and including additional aspects of the social environment.

Despite these limitations, this research is unique in examining prospective associations between the participants' perceived neighborhood social environment and BMI two years later in youth. In addition, to our knowledge, this is the first study to have examined the mediating mechanisms through which the social environment may operate to influence BMI in youth. Including objectively measured behaviors (i.e. MVPA and sedentary time) is a further strength.

## Conclusions

In conclusion, the study findings suggest that neighborhood social networks and social trust/cohesion may be important in preventing overweight and obesity in youth. Considering that there was no evidence for a mediating effect of a range of physical activity behaviors, screen-time or sedentary time, future research investigating the working mechanisms through which the neighborhood social environment exerts its effect on BMI is needed. These studies should include measures of dietary intake and more precise measures of physical activity and sedentary behavior.

## Competing interests

The authors declare that they have no competing interests.

## Authors' contributions

JV conducted the data analysis and drafted the manuscript. MvS, MC and StV assisted with the statistical analysis and study design. DC and JS provided critical comments on the analysis and the manuscript. AT contributed to the analysis and study design. All authors contributed to the interpretation of data and drafting of the manuscript. All authors read and approved the final manuscript.
